# Retraction notice to “Curcumin blunts epithelial-mesenchymal transition of hepatocytes to alleviate hepatic fibrosis through regulating oxidative stress and autophagy” [Redox Biology 36 (2020) 101600]

**DOI:** 10.1016/j.redox.2025.103690

**Published:** 2025-06-04

**Authors:** Desong Kong, Zili Zhang, Liping Chen, Weifang Huang, Feng Zhang, Ling Wang, Yu Wang, Peng Cao, Shizhong Zheng

**Affiliations:** a Chinese Medicine Modernization and Big Data Research Center, Nanjing Hospital of Chinese Medicine Affiliated to Nanjing University of Chinese Medicine, Nanjing University of Chinese Medicine, Nanjing, 210022, China; b Jiangsu Key Laboratory for Pharmacology and Safety Evaluation of Chinese Materia Medica, Nanjing University of Chinese Medicine, Nanjing, 210023, China; c Department of Pharmacology, School of Pharmacy, Nanjing University of Chinese Medicine, Nanjing, 210023, China; d Department of Pharmacology, School of Integral Medicine, Nanjing University of Chinese Medicine, Nanjing, 210023, China; e Department of Oncology, Nanjing Hospital of Chinese Medicine Affiliated to Nanjing University of Chinese Medicine, Nanjing University of Chinese Medicine, Nanjing, 210022, China; f Affiliated Hospital of Integrated Traditional Chinese and Western Medicine, Nanjing University of Chinese Medicine, Nanjing, 210028, China

This article has been retracted: please see Elsevier Policy on Article Withdrawal (https://www.elsevier.com/about/policies/article-withdrawal).

This article has been retracted at the request of the Editors-in-Chief.

The article duplicates significant parts of a paper that had already appeared in Redox Biology, 2017, 11, 322–334 https://doi.org/10.1016/j.redox.2016.12.021, with apparent duplication of six (6) of the DAPI images presented in Fig. 4, panel C of the above mentioned paper with those presented in Fig. 4, panel D of the current paper. In addition, there is duplication of an image in Fig. 1, panel C, with an image in Fig. 7, panel A; and Fig. 2, panel C, has a pair of images with overlapping sections that are ascribed to different conditions. As such, the inclusion of these images in the article represents a misuse of the scientific publishing system. The scientific community takes a very strong view on this matter and apologies are offered to readers of the journal that this was not detected during the submission process.

